# Some Recent Events Connected with the Dental Profession in England

**Published:** 1885-07

**Authors:** W. H. Waite

**Affiliations:** Liverpool, England


### Independent Practitioner.

VOL. VI. JULY, 1885. No. 7.

### Original Communications.

### SOME RECENT EVENTS CONNECTED WITH THE DENTAL PROFESSION IN ENGLAND.

BY W. H. WAITE, D. D. S., LIVERPOOL, ENGLAND.

READ BEFORE THE DENTAL SOCIETY OF THE STATE OF NEW YORK, MAY 14, 1885.

*Mr. President and Gentlemen :*

I should be entirely unworthy of the compliment you have paid me, in allowing me to appear before you, if I did not seize the first opportunity to express my deep sense of your kindness, and the very great pleasure it affords me to meet once, "*in propria persona,*" the members and friends of the New York State Dental Society. Further, this being in a sense a public occasion, I desire, as an Englishman and an English dentist, to utter fraternal greetings towards all American brethren, and to acknowledge my vast indebtedness to them, both for many invaluable suggestions and appliances, which have relieved the burden of daily labor, and also for very many additions to the general store of scientific facts, upon which alone we may safely attempt to erect our theories or regulate our practice.

The fame of American dentistry is world wide, but the high character and generous disposition of many American dentists can be known only to those who enjoy social and professional intercourse with them. Speaking again as an Englishman and an English dentist, I am proud to declare that some of the friends I esteem most highly and love most dearly are American dentists.

Gentlemen, it is exactly ten years at this time since I had the honor of presenting you with some not very flattering aspects of the condition of the dental profession in my country. To-day, it is my pleasing duty to report a rapid and vast improvement. It is said that the darkest hour of the night is the hour immediately before dawn. Certainly the period to which I refer appeared the darkest in the history of our profession in England, but dawn was even then approaching. While many were shaking their heads and saying "Men and brethren, what shall we do?" an honest, large-hearted brother of ours in the town of Stockport (Sidney Wormald by name), had long been revolving the question, and had already set himself to the work of answering it.

In August, 1875, a public meeting of dentists was summoned by his invitation, to assemble in Manchester, the most central city in the north of England, for the purpose of considering what could be done to remedy the glaring evils that had become rampant in the dental world.

Up to this time the dental profession was a body without a head, but afflicted with an enormous development of tail. The Odontological Society of Great Britain had foresworn all but scientific subjects, and there was no apparent means by which the public could be protected from the depredations of uneducated and unprincipled adventurers.

Chaos and apathy divided the honors. Those who might have inaugurated reform remained silent, until the dry bones were shaken by the aforesaid meeting in Manchester.

The outcome of this effort was the formation of a "Dental Reform Committee," composed of representative men from different parts of the country, whose aim was to endeavor to procure some act or legal provision making it compulsory upon all who should hereafter enter upon the practice of dentistry, that they should receive some kind of special education before assuming the title of dentist.

The task thus undertaken was neither easy nor hopeful. The proverbial tardiness of legislation was increased by covert and active opposition. The doctors and the druggists were both alarmed, lest they should be deprived of the innocent amusement of extracting teeth for a small consideration.

Rather than imperil or postpone the whole matter, the executive of the Dental Reform Committee wisely consented not to defraud either the doctors or the druggists of their lucrative and agreeable pleasure, but with a generosity whose wisdom some might question, they went further, and conceded to the aforesaid doctors and druggists the privilege of being allowed, should they so desire, to call themselves "dentists," within the meaning of the schedule.

Eventually, however, in July, 1878, about three years after the Manchester trumpet first sounded the alarm, the "Dentist's Act" passed both houses of Parliament and received the Royal assent.

It may be interesting just to quote the principal clause of this Act, in order that its purpose may be more clearly understood.

"From and after the first day of August, 1879, a person shall not be entitled to take or use the name or title of dentist (either alone or in combination with any other word or words), or of dental practitioner, or any name, title, or description, implying that he is registered under this act, or that he is a person specially qualified to practice dentistry, *unless he is registered* under this Act."

Thus it will be seen that twelve months elapsed after the passing of the act before it came into operation. This period was afforded to enable all existing dentists to register themselves. After the 1st of August, 1879 (with one or two reservations, in behalf of pupils and colonial practitioners), the necessary requirement for registration was that the applicant should be "a licentiate in dental surgery or dentistry of any of the medical authorities." By this means the door of admission to the practice of dentistry in Great Britain was practically closed to Tom, Dick and Harry, until these individuals should have passed through a prescribed curriculum of study, submitted themselves to a thorough examination, and obtained the necessary license.

Thus a great advance is recorded; an advance the import of which, both to the British public and to the Dental profession, cannot be exaggerated; an advance upon the lines of wise and beneficent legislation, the aim of which must ever be to secure "the greatest good to the greatest number."

It would obviously be invidious in a rapid sketch like this to attempt any eulogy, or to pay anything more than a passing tribute of admiration and gratitude. The name of John Tomes is familiar to dentists throughout the world, and it will descend to posterity, associated not alone with laborious investigations in the realms of science, but as the head of this great movement, whereby our calling has been lifted out of degradation into a recognized position. Alongside we must place the well-known and much respected name of James Smith Turner, to whose sleepless watchfulness and untiring energy very much of the rapidity and success of our advancement must be attributed. Thus wisdom and resolute determination were allied, and future generations laid under a perpetual obligation. To these two men, ably supported as they were by the counsel and co-operation of the whole of the committee, belongs the satisfaction of something accomplished in the interest of humanity; not a perfect thing, but as perfect probably as it could be made; not a present boon, but a broad foundation upon which shall arise, as years roll on, a superstructure of encouragement and stimulus to future practitioners, and of comfort and blessing to the general community. All honor to men who labor to such noble purpose.

Now, should we turn aside for a moment, we may notice another event or movement which ran parallel with the efforts of the Dental Reform Committee, though they neither encouraged nor assisted it.

So far back as 1859, permissive power was granted to the College of Surgeons in London, enabling them at their pleasure to examine and confer a license upon dental practitioners. Comparatively few English dentists (200 or 300) availed themselves of this condescension; but a curriculum was carefully prepared, Dental Hospitals began to assume form as schools for students, and some good work was being done, year by year, in the training of young men. The "Dentist's Act" was designed to making this license a *sine qua non* for future practitioners, and if it were allowed to pass without some increased facilities for obtaining the license, an invidious distinction would be created by law between the fortunate possessors of this license and those who, not having been quite awake about the year 1859, had neglected to obtain it for themselves during the years of grace.

The College in London had, with true British dignity, long since closed its doors except to pupils per curriculum, and with real

British obstinacy practically refused every appeal for a re-opening. Again our honest friend at Stockport solved the problem, and applied himself bravely to the task. Another meeting was called in Manchester, in May, 1877, to awaken some interest in this matter, and endeavor, if possible, to induce the College of Surgeons in Ireland, or one of the other licensing bodies of Great Britain, to appoint an examining board for dentists, and issue licenses.* Meetings on this subject were held in several of the principal cities of England. A strong representation was made to the Irish College, and they wisely took the matter into consideration. A clause was inserted in the Dentist's Act, giving authority to each of the licensing bodies to establish a dental department, and immediately upon the passage of the act a considerable number (some 400 or 500) of English dentists of repute, and of established practice, entered themselves for examination in Dublin, and thus obtained what in England it was impossible for them to get, a recognized dental qualification.

Perhaps it should be stated that after a while the English College authorities determined to open their doors once more, with saving restrictions, however, for the maintenance of their dignity. Also the Edinburgh College of Surgeons, together with the Faculty of Physicians and Surgeons of Glasgow, all conspired to multiply facilities for the obtainment of a legitimate qualification.

It cannot be denied that herein we mark another decidedly onward step; not that we are of opinion that a diploma is of great value to the practitioner *per se*, but rather to the public, since the possession of it, if honestly obtained, affords some guarantee of fitness for the duties assumed. In England, however, our profession has hitherto held a very ambiguous position; the number of accredited dentists has been very small, and the growth of public demand for such accreditment was, except in some of the large cities, almost imperceptible. Such a movement as the admission of 400 or 500 practitioners to the ranks of qualified men could not fail to make a deep impression. Hence we find that in all hospital appointments the L. D. S. is now an essential qualification; that in seeking for recommendations people are notably disposed to ask, "Is he qualified ?" and, best of all, in a professional sense, there has

* All the licensing bodies in Great Britain derive their powers from the English Parliament, so that legally they stand on an equal footing.

been these late years a marked and very general increase of good feeling and acknowledged recognition on the part of the medical profession.

The importance of the latter fact can scarcely be measured except by those who are well acquainted with two other collateral facts, viz: first, the very high and deservedly respected social status of the English "doctor," and second (whether for good or ill), the intimate association of the dental with the medical profession, the former being now indeed an acknowledged department of the latter. For a dentist to be recognized at all as above the level of a tinker or a barber is still something in many parts of England, but an immense improvement is rapidly being effected, and this generation shall not pass till our highest hopes in this connection will be fulfilled.

Now, gentlemen, if you are not already weary of "foreign politics," I have yet another item of intelligence which, to American ears, will be more welcome and familiar than either of the foregoing.

The Dentist's Act was passed in the month of July, 1878. The Dental Reform Committee had then performed its functions. The question at once arose, What next? Should the committee resolve itself into its original elements, or should it use the prestige and influence gained by success, for the future providing and guidance of the now legally constituted dental profession?

Much anxious thought and many diverse opinions were given, but manifold good intentions at length culminated in the resolve to endeavor to organize a permanent association, to be modeled somewhat after the fashion of the "British Medical Association," but possessing enough elasticity to accommodate itself readily to new and peculiar conditions.

Accordingly, in the month of March, 1879, a public meeting of dental practitioners, convened by advertisement in the London "Times," was held in Willis's Rooms, London, for the declared object of converting the Dental Reform Committee into the nucleus of a new association. The day was an eventful one in the history of English dentistry. Hitherto there had been far too much of jealousy and mistrust and apathy between differing sections, and particularly as between the metropolitan and provincial members of our body. If success were to attend any future enterprise, there must be mutual conciliation and mutual earnest resolve to work generously and patiently together.

The venerable leader, John Tomes, respected and trusted by all, presided over the meeting, at which between one hundred and two hundred dentists were present, from all parts of the country. Resolutions gratefully recording past labor, and constituting the Reform Committee into a Representative Board were unanimously passed, and with quiet, wistful, perhaps in some instances questioning spirit, but with much hope and self-devotion on the part of many, the new ship was launched, and ere that meeting separated the dental profession in England had a head placed upon its shoulders, a head connected in a thoroughly liberal and representative manner with every part of the body, and there had come into existence the "British Dental Association."*

Not further to detain you by the recital of history, it will be sufficient to say that though this occurred so recently as 1879, there have come into existence no fewer than six branches of the association in different districts of Great Britain, viz :

The Midland Branch, founded 1880.
The Western Counties, founded 1882.
The Scotch Branch, founded 1883.
The Eastern Counties, founded ——.
The West of Scotland, founded 1884.
The Central Branch, founded ——.

All these are exerting a healthy, purifying influence, by furnishing opportunities of intercourse and discussion upon any or every subject connected with the practice of our specialty. Then we have a journal, the particular organ of the association, and the best journal in England. Besides these, a benevolent fund has been established under the auspices of the association, which is already doing excellent service in behalf of helpless widows and orphans.

The establishment of the British Dental Association marks a new era in the dental profession; it is not only an advance, but it is a new departure. Organized upon a thoroughly broad and representative basis, sanctioned by the approval and authority of gov-

---

* The objects for which the association is established are the promotion of dental and the allied sciences, and the maintenance of the honor and the interests of the dental profession. (See Articles of Association, page 1.)

ernment, offering inducements to all and exclusion to none who desire the real elevation of our calling, affording through its various branches occasion for exercise and development of latent power in each section of the country, the British Dental Association promises to exert a mighty renovating influence upon all our future progress.

Though but six years old, the association already embraces a large number of qualified and reputable dental practitioners in England and Scotland, with not a few from the sister Isle. Those who for many years have occupied high positions in London and other large cities have generously supported the association from the first, and, as a matter of fact, the British Dental Association includes (with very few exceptions) all the best dentists in Great Britain.

We want it to be understood that the future position and progress of dental surgery in the British Isles is committed to the care and entrusted to the guidance of the British Dental Association, and that the British Dental Association is the direct, the sole, the responsible representative of dental surgery amongst us.

Gentlemen, my sketch is a very rapid and cursory one; circumstances have rendered it impossible that it should be otherwise, but I am not aware of having omitted any important fact.

To tell you of the self-denial, the unwearying labor, the anxious and sometimes doubtful thoughts, the many bitter discouragements, as well as the pleasurable disappointments attendant upon these several changes, would be an impossible undertaking.

To picture before you the imperfections, the indifference, the selfishness and jealousy still existent, would be now an unprofitable task.

My purpose in this very hasty and imperfect review has simply been to make you acquainted with plain facts, accomplished and present, in the dental profession in England, and, in conclusion, let us hope that whether at home or abroad, wherever our services may be required, the members of the dental profession will ever be found able, willing, and devoted to the noblest of all labor, the relief of human suffering and the increase of human comfort and happiness. Whatever the surroundings or circumstances of our calling may be in particular countries or districts, I am sure we all feel that dental practice in itself affords scope for the highest intellectual power, and the purest moral principle, and that for us all, the wide world over, our standing before men will ever depend, not upon legal enactments, nor combinations for self-defense, but entirely and alone upon individual knowledge and skill, exercised under the control of individual integrity and self-renunciation.

A selfish man cannot long retain the esteem of his fellows, but a simple, steadfast, unflinching purpose, consecrating every power, every resource, to the general well-being of those around, can never fail to exalt the individual, and through him the profession to which he belongs, to the highest rank of public opinion.

---

The members of the Midland Branch of the "British Dental Association," having learned that the Secretary is about to meet the "Dental Society of the State of New York" at their annual meeting, desire to convey through him cordial expressions of esteem and fraternal greeting toward the dental profession throughout the United States of America.

*President*, H. BLANDY, (Nottingham.)
*Treasurer*, S. WORMALD, (Stockport.)
*Secretary*, W. H. WAITE, (Liverpool.)

